# Hepatic Encephalopathy— A Serious Complication of Alcoholic Liver Disease

**Published:** 2003

**Authors:** Roger F. Butterworth

**Affiliations:** Roger F. Butterworth, Ph.D., D.Sc., is scientific director of the Neuroscience Research Unit at CHUM (Hôpital Saint-Luc), and professor of medicine at the University of Montreal, Montreal, Canada

Alcohol’s harmful effects on liver cells not only interfere not only with the normal functioning of the liver but also impact distant organs, including the brain. Prolonged liver dysfunction resulting from excessive alcohol consumption can lead to the development of a serious and potentially fatal brain disorder known as hepatic encephalopathy (HE). Patients with HE suffer from sleep disturbances, changes of mood and personality, severe cognitive effects (e.g., a shortened attention span), psychiatric conditions such as anxiety and depression, as well as motor disturbances, including motor incoordination and a type of flapping tremor of the hands called asterixis. In the most serious cases, the patients no longer respond to external stimuli and may fall into a coma (i.e., hepatic coma), which can be fatal.

Analyses of brain tissue of HE patients found characteristic changes in the structure of supporting cells known as astrocytes rather than obvious destruction of nerve cells (i.e., neurons). Astrocytes are large star-shaped cells, distributed throughout the brain, that help maintain the proper composition of the fluid surrounding the neurons. For example, astrocytes take up brain chemicals (i.e., neurotransmitters) that are released by neurons, and minerals such as potassium, which are generated and secreted during the brain’s energy metabolism. In addition, astrocytes eliminate some substances that are toxic to neurons (i.e., neurotoxic). The proper functioning of the astrocytes and their interactions with the neurons are essential to brain function. Patients with HE frequently have pairs and triplets of abnormal astrocytes with a characteristic structure known as Alzheimer type II astrocytosis, in which the astrocytes’ nuclei are enlarged and glassy-looking. This glassy appearance is caused by the fact that the DNA and its associated proteins are confined to the edges of the nuclei, rather than distributed throughout them. Alzheimer type II astrocytes also exhibit other physiological and functional abnormalities.

Diagnosing HE in alcoholic patients is difficult because no single clinical or laboratory test can conclusively establish the diagnosis. Patients frequently are misdiagnosed, particularly in the early stages of HE, when symptoms such as euphoria, anxiety, depression, and sleep disorders occur that are common to a number of psychiatric conditions. In addition, whether—and to what extent—a patient shows each of these symptoms depends on fluctuations in the patient’s medical status or diet. Diagnosis also is hindered because HE can be triggered or exacerbated by a medical procedure known as the transjugular intrahepatic stent shunt (TIPS), which commonly is used to treat alcoholic patients who experience elevated blood pressure in the portal vein that transports blood to the liver. By redirecting blood flow around the liver, the TIPS procedure is intended to alleviate this condition and prevent complications such as gastrointestinal bleeding and accumulation of fluid in the abdomen (i.e., ascites).

## Relationships Between the Liver and the Brain

Normal brain functioning depends on several aspects of normal liver functioning. For example, the liver supplies certain nutrients to the brain that the brain itself cannot produce. The liver also cleanses the blood of substances that could damage brain cells (i.e., neurotoxins). Although the brain is protected from many neurotoxic substances by the blood–brain barrier—a property of blood vessels in the brain that prevents passage of many compounds from the blood into the brain tissue— certain neurotoxins can penetrate that barrier. These substances—which include ammonia, manganese, and other chemicals—can enter the brain at least to some extent unless they are effectively removed from the blood by the liver.

In patients with fibrosis or cirrhosis (whether caused by excessive alcohol consumption or factors such as viruses or toxins), the liver loses its capacity to remove toxic substances from the blood because the number of functional liver cells (i.e., hepatocytes) has decreased. Moreover, in these patients some of the blood that normally flows through the portal vein into the liver for cleansing is diverted directly into the general circulation without first passing through the liver, a phenomenon known as portal-systemic shunting. As a result, the shunted blood is not detoxified and blood levels of toxic substances rise. Persistently elevated neurotoxin levels damage brain cells and the patients begin to develop HE.

In fact, studies involving neuropsychological tests have found that although alcohol’s direct effects on the brain also cause cognitive deficits and brain damage in alcoholics, HE is a major contributing factor to cognitive dysfunction in alcoholics with severe liver disease. In these studies, alcoholic patients with cirrhosis had significantly lower scores on learning and memory tests than did alcoholics without cirrhosis, indicating that liver dysfunction is associated with more extensive brain dysfunction in these patients (Tarter et al. 1993).

## Mechanisms Leading to HE

Researchers have gained a better understanding of the mechanisms leading to HE in patients with alcoholic liver disease by using neuroimaging and spectroscopic techniques that permit them to study the metabolism and functions of specific brain regions in living patients. These studies have confirmed the contributions of at least two neurotoxic substances, ammonia and manganese, to the development of HE.

### The Role of Ammonia

Some of these investigations employed positron emission tomography (PET), a technique used to examine the metabolic activity of various body regions, including the brain, by monitoring the transport and breakdown of radioactively labeled molecules using sophisticated detection devices. Some PET studies of alcoholic patients have assessed ammonia uptake and metabolism in the brain. In cirrhotic patients with mild HE, PET analyses using radioactive ammonia have revealed significant increases in the amount of ammonia taken up and metabolized in the brain (Lockwood et al. 1991). In particular, a variable called the permeability–surface area product (PS), a measure of how much ammonia can enter the brain from the general circulation, increases as cirrhotic patients start to develop HE. When the PS increases, a greater proportion of the ammonia in the general circulation can enter the brain.

The brain has only a limited capacity to remove any ammonia coming in because of the increased PS. The only way to eliminate any ammonia that has reached the brain cells is through a reaction mediated by an enzyme called glutamine synthetase, which is found in the astrocytes. This enzyme combines a molecule of the amino acid glutamate with a molecule of ammonia to form the amino acid glutamine. In patients with HE, the amounts of glutamine formed in the brain are correlated with the severity of the disease, indicating that the brain is exposed to increasing levels of ammonia as the disease progresses (Lockwood et al. 1997; Butterworth 2002).

Ammonia adversely affects both neurons and astrocytes. Because the enzyme that eliminates ammonia in the brain is present only in astrocytes, neurons are virtually defenseless against increased ammonia concentrations and therefore are likely to suffer ammonia-related damage. For example, ammonia has deleterious effects on nerve signal transmission that is mediated by numerous neurotransmitter systems (Szerb and Butterworth 1992) and impairs the brain’s energy metabolism. In addition, ammonia can alter the expression^1^ of various genes that encode key brain proteins involved in the brain cells’ energy production, structure, and cell-to-cell interactions. These alterations in gene expression may account for some of the changes in neurotransmitter activity and astrocyte structure observed in HE patients.

### The Role of Manganese

Researchers also have used magnetic resonance imaging (MRI) to analyze changes in the brains of alcoholics. This technique generates images based on differences between tissues in water content as well as in the content of other molecules that respond to a magnetic field. MRI analyses have found that more than 80 percent of alcoholics with cirrhosis show regions of abnormally high signal intensity (i.e., signal hyperintensities), primarily in a brain area called the globus pallidus, which is involved in control of motor function (Lockwood et al. 1997; Spahr et al. 2000). The intensity of these signals correlates with the presence of certain signs and symptoms of impaired motor function but not with the patients’ performance on tests assessing global encephalopathy and cognitive functioning.

Additional analyses have determined that hyperintense MRI signals in the globus pallidus are probably caused by manganese deposits in that region (Lockwood et al. 1997). Indeed, studies using brain tissue from alcoholic cirrhotic patients who died from HE have revealed manganese levels in the globus pallidus that were up to seven times higher than manganese levels in subjects without cirrhosis (Butterworth et al. 1995). Manganese normally is eliminated by the joint actions of the liver, gallbladder, and bile ducts (i.e., the hepatobiliary system), but patients with chronic liver failure have elevated manganese concentrations in the blood. As a result, the metal can enter the brain and be deposited in the globus pallidus and associated brain structures, where it particularly affects the actions of certain proteins (i.e., receptors) that interact with the neurotransmitter dopamine. This effect is demonstrated by the fact that dopamine receptors are altered in the brains of alcoholic cirrhotic patients who died in a hepatic coma (Mousseau et al. 1993). In addition, manganese induces Alzheimer type II changes that interfere with the functioning of astrocytes. Thus, manganese deposits in the globus pallidus may account for both the motor symptoms and the structural changes in astrocytes that are characteristic of HE.

## Treatment of Patients With HE

Researchers and clinicians are exploring various approaches to preventing HE in patients with alcohol-induced chronic liver failure or to ameliorating its consequences. These approaches include the following:

- *Strategies to lower ammonia levels.* One approach— administering certain sugar molecules (e.g., lactulose) or antibiotics (e.g., neomycin)—reduces the production of ammonia in the gastrointestinal tract. Other strategies are intended to increase the conversion of ammonia into harmless molecules outside the brain—for example, by treating the patients with an agent called L-ornithine L-aspartate, which helps to incorporate ammonia into the amino acid glutamine in the skeletal muscle—and to bolster the residual ability of the patient’s cirrhotic liver to eliminate ammonia as urea.
- *Neuropharmacological strategies.* These approaches involve using neuroactive drugs to counteract ammonia’s harmful effects on neurotransmitter systems in the brain. This type of treatment is in its infancy, however, because researchers have not yet identified the precise nature of the neurotransmitter systems that contribute to the development of HE or are affected by the condition.
- *Liver-assist devices.* These machines, or “artificial livers,” are dialysis systems composed of columns that are filled with hepatocytes, a protein called albumin, charcoal, or combinations thereof. The patient’s blood is circulated through these columns to remove the toxins. In initial studies, patients treated with an albumin-based system showed lower amounts of ammonia circulating in the blood as well as improvements in the severity of their encephalopathy (Mitzner and Williams 2003).
- *Liver transplantation.* This approach is widely used in alcoholic cirrhotic patients with end-stage chronic liver failure. In general, implantation of a new liver results in significant improvements in cognitive function in these patients (Arria et al. 1991) and corrects the excessive ammonia levels as well as the MRI signal hyperintensities that result from manganese deposits found in patients with HE (Pujol et al. 1993).

## Summary

HE is a serious complication of alcoholic liver disease that contributes to cognitive dysfunction in chronic alcoholic patients. In patients with HE, the damaged liver can no longer remove neurotoxic substances such as ammonia and manganese from the blood. As a result, these molecules may enter the brain, where they can exert a variety of harmful effects that interfere with normal neurotransmitter activity, impair motor functions, and cause structural alterations in the astrocytes. To prevent or treat HE in alcoholic patients with cirrhosis, physicians currently rely primarily on strategies to lower blood ammonia concentrations as well as on liver transplantation in patients with end-stage liver disease; new approaches also are also being investigated.

## Footnotes

1 The term “gene expression” refers to the entire process of converting the genetic information encoded in a gene into a protein product.
